# A systematic review of interventions to mitigate radiotherapy-induced oral mucositis in head and neck cancer patients

**DOI:** 10.1007/s00520-020-05548-0

**Published:** 2020-09-05

**Authors:** Catrina Davy, Sharron Heathcote

**Affiliations:** 1 London, UK; 2 University Cardiff, Wales, United Kingdom

**Keywords:** Radiotherapy, Radiation therapy, Head and neck cancer, Oral mucositis

## Abstract

**Background:**

Oral mucositis is a debilitating consequence of radiotherapy in patients with head and neck cancers. Radiation-induced oral mucositis (RIOM) can cause pain and weight loss, reduce quality of life and affect treatment outcomes.

**Methods:**

A systematic review was undertaken to identify and examine the efficacy of low-cost interventions to mitigate RIOM and to develop clinical guidelines based on the evidence.

**Results:**

The author identified three interventions: benzydamine hydrochloride mouth rinse (BHM), honey and oral glutamine (OG). The search identified twenty-four studies in total. Four studies examined BHM; all findings were favourable, although only one had moderate methodological quality, and the rest were low. The product was poorly tolerated by some participants in one study. Twelve studies examined honey. Eleven of these had favourable results; two studies had moderate methodological quality, and the rest were low. Eight studies examined OG. Six of these had favourable results; two studies had moderate methodological quality, and the rest were low.

**Conclusion:**

The author cannot recommend BHM to mitigate RIOM due to the overall low quality of the studies and poor tolerance to the product. The author cannot recommend honey to mitigate RIOM due to weak evidence supporting the intervention. The author can recommend OG to mitigate RIOM. There is a need for high-quality studies with a consensus of the methodology to reduce heterogeneity and examination of the cost-effectiveness of the interventions.

## Introduction

Oral mucositis (OM) is a painful condition, characterised by ulcers [1]. Rapid cell division in the oral tract makes mucosal cells particularly sensitive to damage by irradiation [2]. OM commonly occurs in head and neck cancer patients (HNCPs) who have had radiotherapy (RT). It can affect up to 100% of HNCPs [3], and it is therefore a significant problem for this group. Radiation-induced oral mucositis (RIOM) can have a detrimental effect on patients’ functioning and quality of life (QoL): The painful inflammation and ulceration may affect patients’ ability to eat, drink and talk [4]. It may cause nutritional deficiencies affecting patients’ energy which can cause weight loss [4]. If severe RIOM occurs, it can affect patients’ health outcomes due to missed radiotherapy treatments; in fact, RIOM is the most likely side effect of RT to the oral region, causing limited RT doses [5].

The model for OM pathogenesis includes five stages: firstly, direct cell damage to the DNA, followed by tissue damage to the submucosa and basal epithelium, leading to inflammation then ulceration of the tissue (where bacteria then cause even more inflammation) and healing as the final stage [6].

Grade 1 RIOM generally starts after approximately 2 weeks of RT, with grade 3 RIOM generally occurring after approximately 3 weeks. Commonly, RIOM peaks 2 weeks after treatment is completed and is resolved 8 weeks after that [7].

Effective interventions are essential to mitigate RIOM; improve patients’ functioning, QoL, and health outcomes; and limit weight loss.

National guidelines for oral care for patients at risk of OM were determined by two organisations in the United Kingdom (UK): the UK Oral Mucositis in Cancer Group (UKOMCG), updated in June 2019, and the Royal College of Surgeons of England and the British Society for Disability, updated in 2018. However, it is unclear how these organisations selected the studies on which they based their recommendations. Also, some of these studies were not contemporaneous. The search for contemporaneous studies in this review identified fifty-eight interventions for the management of RIOM in the last 5 years. For the majority of the interventions, there were few studies conducted with small sample sizes making it difficult to establish efficacy.

National Institute for Health and Clinical Excellence (NICE) guidelines, published in May 2018, recommended low-level laser therapy (LLLT) as an effective intervention for OM. However, the implementation of this intervention in a service may incur high set-up costs for equipment and training of staff. Therefore, this study focussed on examining the efficacy of low-cost interventions, which incur no set-up costs.

The aim of this study was to conduct a systematic review (SR) of contemporaneous studies to examine the efficacy of low-cost interventions to mitigate RIOM.

## Methods

### Study design

The study was designed to establish the efficacy of interventions to mitigate RIOM in HNCPs undergoing RT through a SR of contemporaneous evidence.

### Eligibility criteria

#### Inclusion criteria

Studies that fitted the following criteria were included:

- Randomised controlled trials (RCTs), SRs and meta-analyses (MAs)
- Patients receiving RT, with or without chemotherapy (CT), for head and neck cancers
- Interventions where there had been four or more studies conducted
- Studies conducted in the last 5 years (from 2014 to 2019)
- Studies in English language
- Studies of adults

#### Exclusion criteria

Studies were excluded if they fitted the following criteria:

- Studies where full text was not available
- Studies where the interventions had added costs for equipment and training

### Search strategy

The search for literature was conducted using the following databases: Amed, CINHL, Cochrane Library, EMBASE, EMCARE, Google Scholar, Medline via ovid and PubMed. The reference lists of the identified studies were also examined to find additional studies that fit the criteria that were not found through the database search.

**Fig. 1:**
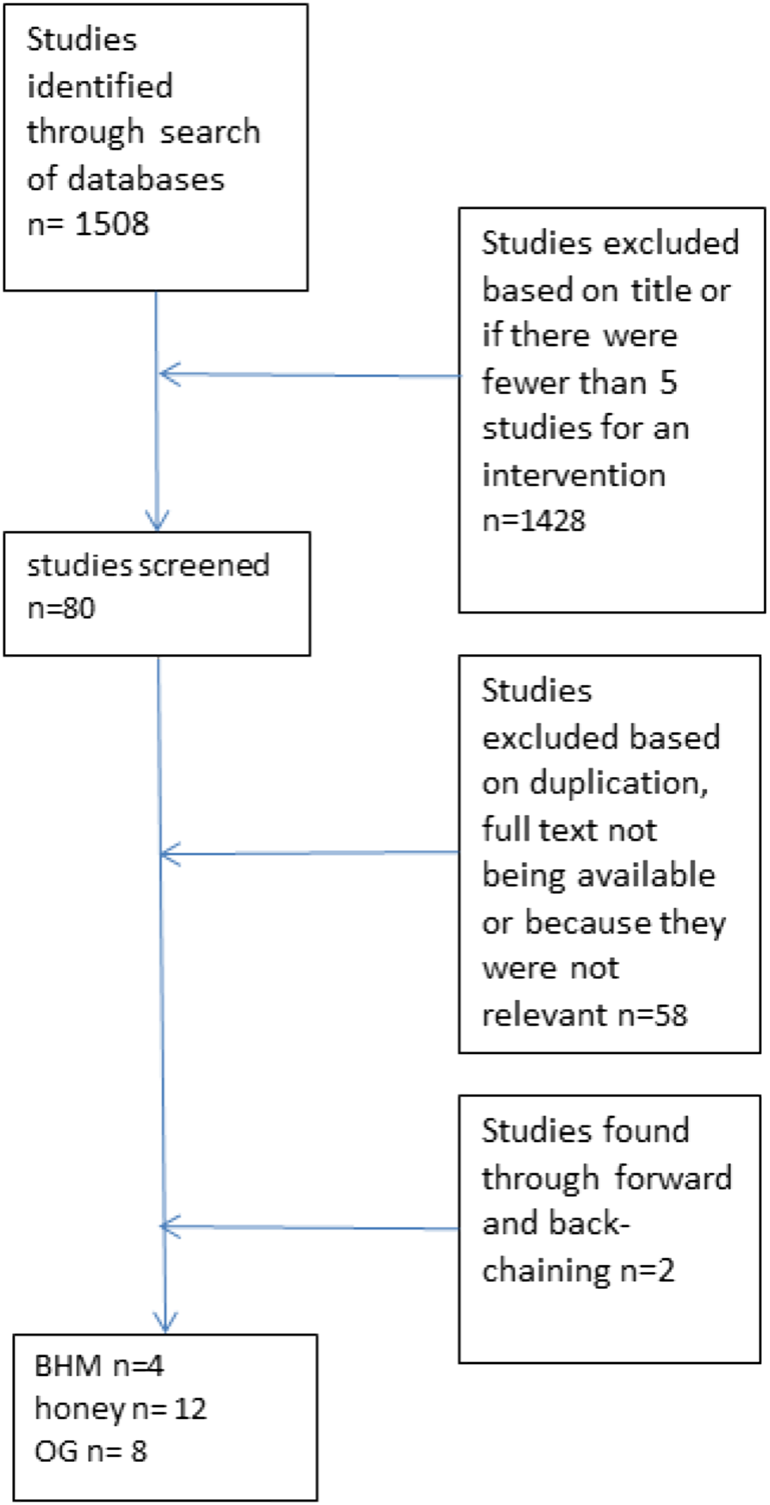
Flow chart showing the search strategy adopted in this review

Keywords used in the search were “Radiotherapy” or “radiation therapy” and “oral mucositis” or “mucositis”. The following Boolean operators were utilised: AND and OR. Figure 1 shows the search strategy adopted in this SR.

### Outcomes

The primary outcome measure was OM grade using any appropriate assessment scale, recorded in any format (for example incidence of severe OM, onset of OM, duration of OM) or OM pain (measured using a visual analogue scale or numerical rating scale). The following OM assessment tools were identified: World Health Organization (WHO) OM assessment tool; Radiation Therapy Oncology Group (RTOG) OM grading system; Oral Mucositis Assessment Scale (OMAS) and the National Cancer Institute Common Terminology Criteria for Adverse Events (NCI CTCAE). All secondary outcome measures were included.

### Assessment of methodological quality and quality of evidence

The studies’ methodologies were appraised utilising Critical Appraisal Skills Programme (CASP) checklist for RCTs and SRs and recorded on Excel sheets. The quality of the evidence for all studies was assessed using Harbour and Miller’s (2001) Hierarchy of Evidence. The assessments were conducted by the author.

### Data collection

Data from the studies was collected and recorded on standardised Excel forms by the author. The data extracted included author, year, title, aim of the study, study design, sample size, inclusion and exclusion criteria, randomisation method, intervention, control, details of cancer treatment, primary and secondary outcome measures, results and conclusion.

### Risk of bias across studies

The author considered the risk of bias across the studies.

### Translation of results into clinical guidelines

The findings of the review were applied to the GRADE Evidence to Decision (EtD) framework [8] to inform clinical guidance for the mitigation of RIOM.

## Results

### Study selection

Initially, the search identified 1508 studies. One thousand four hundred eighty-six studies were excluded because they did not meet the set criteria or were duplications. A search of reference lists identified two more studies. In total, twenty-four studies met the inclusion criteria.

### Study characteristics

A summary of the studies’ characteristics and results are in Tables 1 and 2.

**Table 1:** Summary of the RCTs’ characteristics and results

Author	Study design	Sample size	OM assessment tool	Primary outcome measures	Secondary outcome measures	Intervention	Control	Type of oncology treatment	Main result
Benzydamine hydrochloride mouth wash (BHM)									
Sheibani (2015)	Single-centre double-blind placebo–controlled RCT	51	Own assessment tool	Mean mucositis score	Weight, vital signs, blood count, electrolytes and renal function test	Rinse with 15 ml of BHM for 2 min 4–8 times per day from 1 day prior to RT to 2 weeks after	Placebo	RT or CRT (> 50 Gy)*	Weeks 4–7: *p* = 0.01
Diwan and Meshram (2016)	Single-centre randomised, prospective, comparative study	80	RTOG	Incidence of OM at 1 month post RT	None	Rinse BHM for at least 5 min, three times a day	2% povidone iodine rinses	RT (60–66 Gy)	10.3% of patients in BHM group had grade 2 OM compared with 15.7% in the control group. 61.5% of patients in BHM group had grade 0 OM compared with 44.8% in the control group
Rastogi et al. (2017)	Single-centre RCT	120	WHO and NCI CTCAE vs 4.0	Incidence of grade 3 OM	Number of patients requiring feeding tubes, IV fluid and hospital admissions	Rinse 10 ml of BHM for at least 1 min 4–6 times a day	Saline	RT or CRT (> 50 Gy)**	RT alone WH0: *p* = 0.038, CTCAE: 0.043; CRT WHO: *p* = 0.091, CTCAE: *p* = 0.30
Chitapanarux et al. (2018)	Double-blind, multi-centre RCT	60	OMAS	Median total OMAS score	Pain, analgesia, artificial saliva supplements, anti-infection medications, number of patients requiring feeding tubes or nutritional support, hospital admissions, treatment interruptions	Rinse 15 ml of BHM for 2 min four times a day, time from before start of RT to 2 weeks after	Sodium bicarbonate	CRT (≥ 60 Gy)***	*p* < 0.001
Honey									
Hawley et al. (2014)	Double-blind multi-centre placebo-controlled RCT	106	RTOG, WHO and OMAS	Incidence of severe OM	Weight, pain, QoL	5 ml of honey rinsed for at least 30 s then swallowed, 4 times a day	Placebo gel	RT or CRT (> 50 Gy) ^X^	*p* = 0.4126
Samdariya et al. (2015)	Singe-centre RCT	78^+^	N/A	Mean pain score (VAS)	None	20 ml of honey 15 min before, then 15 min and 6 h after RT	Standard care	CRT (≥ 66 Gy)****	*p* < 0.001
Jayalekshmi et al. (2016)	Single-centre, single-blind RCT	28	RTOG	Incidence of severe OM	None	15 ml of natural honey rinsed then slowly swallowed 3 times/day throughout course of RT	Water	RT or CRT (≤ 64 Gy)****	Weeks 4–6: *p* < 0.01
Amanat et al. (2017)	Single-centre RCT	82	RTOG	Incidence of grade 3 or 4 OM	None	20 ml of honey 15 min before and after RT and before sleep at night throughout RT	Saline	RT (60–70 Gy)****	Grade 3 OM, *p* = 0.016 and grade 4 OM, *p* = 0.032
	Study design	Sample size	OM assessment tool	Primary outcome measures	Secondary outcome measures	Intervention	Control	Type of oncology treatment	Main result
Rao et al. (2017)	Single-centre, single-blind RCT	50	RTOG	Incidence of OM	RT interruptions, weight loss, tumour response	Honey 1 h before and 2 and 6 h after RT	Povidone iodine	RT or CRT (62–70 Gy) ^X^	Incidence of grade 3 OM at week 7 *p* = 0.2; incidence of grade 3 OM at week 3 of RT *p* = 0.03 and at week 7 *p* = 0.2
Charalambous, et al. (2018)	Single-centre RCT	72	RTOG	OM grade	Weight, QoL, oral problems (eating, drinking, swallowing, mouth and throat pain)	20 ml of honey mixed in 100 ml water. Gargle product 15 min before RT and 15 min and 6 h post RT	Saline	RT or CRT (50–60 Gy)*****	*p* < 0.001
Oral glutamine									
Chattopadhyay et al. (2014)	Single-centre RCT	70	WHO	Incidence of severe OM	Mean duration and mean onset of OM	10 g of OG in 1000 ml water 2 h prior to RT	Standard care	RT or CRT (RT dose not recorded) ^X^	Grade 3 OM *p* = 0.02; grade 4 OM *p* = 0.04
Tsujimoto et al. (2015)	Single-centre, double-blind RCT	40^++^	NCI CTCAE vs 3.0	Severity of OM (maximal OM grade)	Onset and duration of OM, pain, incidence and duration of opioid use, total opioid dose, incidence of nutritional supplementation	10 mg of OG 3 times a day throughout course of CRT	Placebo	CRT (post-op = 66 Gy or 70 Gy) ^X^	*p* = 0.005
Pattanayak et al. (2016)	Single-centre RCT	162	Not described	Severity of OM	Onset of OM, dysphagia, nausea, oedema, cough, pain, analgesic use, number of patients requiring feeding tubes	15 g of OG rinsed then swallowed twice a day throughout treatment	Standard care	CRT (70 Gy)	*p* < 0.05
Lopez-Vaquero et al. (2017)	Single-centre, double-blind RCT	50	NCI CTCAE vs 3.0	Incidence of clinical OM at 6th week after RT	Incidence of functional OM, onset of OM, cervicofacial dermatitis, pain, weight loss	10 g of OG distributed in meals 3 times a day	Placebo (maltodextrin)	RT or CRT (post-op = 66 Gy or 70 Gy) ^X^	*p* = 0.324
Huang et al. (2019)	Single-centre, double-blind RCT	71	NCI CTCAE vs 4.03	Severity of OM	Grade of dermatitis, treatment interruptions, incidence of opioid use, BMI	10 g of l-glutamine and 5 g of maltodextrin three times a day from 1 week before RT to 2 weeks afterwards	Placebo (15 g maltodextrin)	RT or CRT (post-op = 66Gy or 70Gy)*****	OR = 0.3; 95% CI = 0.05–1.67; *p* = 0.169
Pathak et al. (2019)	Single-centre RCT	60^+++^	NCI CTCAE vs 4.03	Incidence and severity of OM	Onset of OM, dysphagia, weight loss, treatment interruptions, number of patients requiring feeding tubes fitted	10 g of OG with water 2 h before RT, 5 days a week throughout course of RT	Standard care	CRT (70 Gy)*****	*p* < 0.001

Conventional RT delivered except for the following: *conventional or cobalt; **conformal; ***conventional, conformal or IMRT; ****cobalt; *****IMRT only; ^X^not recorded

Studies included all H&N cancer types except for the following: ^+^oral cavity, oropharynx, hypopharynx, larynx; ^++^hypopharynx, larynx, oropharynx, nasopharynx; ^+++^oropharynx, larynx

**Table 2:** Summary of the SR and MA characteristics and results

Author	Study design	Sample size	OM assessment tool	Primary outcome measures	Secondary outcome measures	Intervention	Control	Type of oncology treatment	Main result
Honey									
Cho et al. (2015)	MA	476	Any	Incidence grades 3 and 4 OM, onset to OM, mean OM grade	Bacterial colonisation, fungal colonisation, pain requiring analgesics, weight loss	Honey	Placebo or standard care	RT or CRT	Incidence of severe OM: OR = −1.94, 95% CI = −2.88–− 1.0, *p* < 0.001
Co et al. (2016)	SR and MA	244	WHO, RTOG, OMAS	Peak severity of OM	Onset to OM, RT interruptions, weight loss	Honey	Any intervention defined as standard care	RT or CRT	RR = 0.45, 95%CI = 0.09–2.21
Xu et al. (2016)	SR and MA	381	WHO, RTOG, OMAS	Incidence of OM	None	Honey	No treatment or single-factor intervention	RT, CT or CRT	Studies included in MA: RR = 0.35 95%CI = 0.18–0.70, *p* = 0.003
Yang et al. (2019)	SR and MA	740	RTOG, WHO and NCI NCTCAE	Incidence of moderate–severe OM	Onset to time of OM, swallowing diary, fungal colonisation, bacterial colonisation, analgesic use	Honey	Placebo or usual care	CT, RT or CRT	OR 0.22; 95%CI 0.10–0.47)
Münstedt et al. (2019)	SR	1192	Any	OM grade	Any	Honey	Any	RT or CRT	Studies using Manuka honey (*n* = 4) did not benefit OM; studies using conventional honey (*n* = 13) did benefit OM
Liu et al. (2019)	MA	1276	RTOG, WHO and NCI NCTCAE	Incidence of intolerable OM, number of OM lesions	Duration of recovery time, QoL	Honey	Unclear	CRT	RR = 0.18, 95% CI = 0.09–0.41
Oral glutamine									
Leung and Chan (2016)	MA	234	any	Risk of developing grade 3 or 4 OM	None	OG	Not specified	RT or CRT	RR = 0.17; 95%CI = 0.06–0.47
Sayles et al. (2016)	SR	237	Any	Incidence and severity of OM	Onset to OM, maximum OM grade, number of patients requiring feeding tubes, number of patients requiring supplemental nutrition, duration of opioid use	Vidal-Casariego et al. (2013): 30 mg of OG once a day; Chattopadhay et al. (2014) and Tsujimoto et al. (2015)—see above	Vidal-Casariego et al. (2013): standard care; Chattopadhay et al. (2014) and Tsujimoto et al. (2015)—Table 1	RT or CRT	All 3 studies found statistically significant results favouring OG

The interventions identified, where there were at least four studies, were benzydamine hydrochloride mouth rinse (BHM; RCT, *n* = 4), honey (SR or MA, *n* = 6; RCT, *n* = 6) and oral glutamine (OG; SR or MA, *n* = 2; RCT, *n* = 6). A total sample size for each intervention was BHM (*n* = 311), honey (*n* = more than 3985) and OG (*n* = 924).

Five out of the 16 RCTs used a placebo as control. Other controls used were standard care (*n* = 4), saline (*n* = 3), povidone iodine rinse (*n* = 2), sodium bicarbonate (*n* = 1) and water (*n* = 1). The most commonly used OM assessment tool was the RTOG OM assessment tool (*n* = 10) followed by WHO OM assessment tool (*n* = 9) and NCI CTCAE (*n* = 9) then OMAS (*n* = 4). One study utilised a non-validated OM assessment tool; two RCTs utilised more than one validated OM assessment tool; and one study did not describe how OM was assessed.

OM was presented in twelve ways: incidence of severe OM (*n* = 18), onset of OM (*n* = 9), mean OM grade (*n* = 4), mean maximum OM grade (*n* = 4), duration of OM (*n* = 3), incidence of OM (*n* = 3), reduction of OM (*n* = 2), median OM (*n* = 1), OM recovery time (*n* = 1), number of OM lesions (*n* = 1), functional OM (*n* = 1), mucositis grade at 1 week (*n* = 1). Twenty-nine secondary outcomes were measured. The most common were pain (*n* = 10), weight loss (*n* = 8), treatment interruptions (*n* = 8), number of patients requiring feeding tubes (*n* = 5), number of patients requiring analgesia use (*n* = 4) and quality of life (*n* = 4). The least commonly utilised secondary outcomes measured by only one of the identified studies were number of patients requiring IV fluids, artificial saliva or anti-infection interventions; number of patients who developed dysphagia, nausea, cough or oedema; duration of opioid use; vital signs; blood counts; electrolytes; and renal function.

### Synthesis of results

#### Benzydamine hydrochloride mouth rinse

##### Primary outcome measure (oral mucositis measures)

Two out of the four studies [9, 10] measured incidence of severe (grades 3 and 4) OM. Both found a statistically significant reduction in severe OM in the BHM group. One study [11] measured the mean OM grade and found a statistically significant reduction in the BHM group in weeks 4 to 7 of RT. One study [12] measured the median OM and found a statistically significant reduction in the BHM group. The same study measured the mean maximum OM grade and found a lower OM grade in the BHM group; the statistical significance was not calculated.

##### Secondary outcome measures

Three studies measured treatment interruptions; one study [10] found statistically significantly fewer treatment interruptions in the BHM group receiving RT alone but not in the group receiving chemoradiotherapy (CRT). One study [11] found fewer treatment interruptions but did not calculate statistical significance, and one study [12] found no statistically significant difference between the groups. Two studies measured the number of participants who required feeding tubes fitted. One study [10] found statistically significantly fewer participants in the BHM group, receiving RT alone, required feeding tubes fitted. They found no statistically significant difference between the groups receiving CRT. The second study [12] did not find a statistically significant difference between the groups.

Only one study [12] recorded adverse events (AEs) and found that 6.75% of participants in the BHM group were unable to tolerate the full strength of BHM due to a burning sensation in the mouth.

##### Quality of studies

A summary of the critical appraisal of the RCTs is in Table 3 and SRs and MAs in Table 4.

**Table 3:** Critical appraisal of RCTs using the Critical Appraisal Skills Programme

Author	Was the assignment of patients randomised?	Were all patients accounted for at its conclusion?	Were patients and personnel “blind” to treatment?	Were the groups similar at the start of the trial?	Were the groups treated equally?	How large was the treatment effect?	How precise was the estimate of the treatment effect?	Can the results be applied to the local population?	Were all clinically important outcomes considered?	Are the benefits worth the harms and costs?	Quality of evidence (Harbour and Miller 2001)
BHM											
Sheibani et al. (2015)	Yes	Unclear	Participants and staff	Yes	Yes	Only significant benefit weeks 4–7	Unclear—95% CI not recorded	No	No	Yes	1−
Diwan and Meshram (2016)	Yes	No	No	Unclear	Yes	No significant benefit	Unclear—95% CI not recorded	No	No	Yes	1−
Rastogi et al. (2017)	Yes	Unclear	No	No—tumour stage different	Yes	No significant benefit	Unclear—95% CI not recorded	No	No	Yes	1−
Chitapanarux et al. (2018)	Yes	Yes	Participants and staff	Yes	Yes	Significant benefit	Unclear—95% CI not recorded	Yes	Yes	No—poor tolerance	1+
Honey											
Hawley et al. (2014)	Yes	Yes	Participants and staff	No—age and diabetic status different	Yes	No significant benefit	Unclear—95% CI not recorded	No	No	No—poor tolerance	1−
Samdariya et al. (2015)	Yes	No	No	Yes	Yes	Significant benefit	Unclear—95% CI not recorded	No	No	Yes	1−
Jayalekshmi et al. (2016)	Yes	Unclear	Participants	No—number of patients receiving RT and CRT different	Yes	Only significant benefit weeks 4–6	Unclear—95% CI not recorded	No	No	Yes	1−
Amanat et al. (2017)	Yes	Yes	No	No—different in genders	Yes	Significant benefit	Unclear—95% CI not recorded	No	No	Yes	1−
Rao et al. (2017)	Yes	No	Investigators	No—more T1 patients in treatment group	Yes	No significant benefit	Unclear—95% CI not recorded	No	No	Yes	1−
Charalambous, et al. (2018)	Yes	No	Assessors	Yes	Yes	Significant benefit	Unclear—95% CI not recorded	No	No	Yes	1−
Oral glutamine											
Chattopadhyay et al. (2014)	Yes	Yes	No	Yes	Yes	Significant benefit	Unclear—95% CI not recorded	No	No	Yes	1−
Tsujimoto et al. (2015)	Yes	No	Participants and clinical staff	Yes	Yes	Significant benefit	Unclear—95% CI not recorded	No	No	Yes	1−
Pattanayak et al. (2016)	Yes	Yes	No	No—different for tumour types	Yes	Significant benefit	Unclear—95% CI not recorded	No	No	Yes	1−
Lopez-Vaquero et al. (2017)	Yes	Yes	Participants and clinical staff	Yes	Yes	No significant benefit	Unclear—95% CI not recorded	No	No	Yes	1−
Huang et al. (2019)	Yes	Yes	Patients and clinical staff	Yes	Yes	No significant benefit	Precise—95% CI recorded	No	Yes	Yes	1+
Pathak et al. (2019)	Yes	No	No	Yes	Yes	Significant benefit	Unclear—95% CI not recorded	No	Yes	Yes	1−

**Table 4:** Critical appraisal of SRs and MAs using the Critical Appraisal Skills Programme

Author	Do you think all the important, relevant studies were included?	Did the review’s authors do enough to assess quality of the included studies?	If the results of the review have been combined, was it reasonable to do so?	What are the overall results of the review?	How precise are the results?	Can the results be applied to the local population?	Were all important outcomes considered?	Are the benefits worth the harm and costs?	Quality of evidence (Harbour and Miller 2001)
Honey									
Cho et al. (2015)	No—only Medline, Scopus and Cochrane searched	Yes—Cochrane risk of bias tool	No—different products used as control	Significant benefit	Yes—95% CI documented	Yes	No	Yes	1−
Co et al. (2016)	Yes	Yes—CASP	Yes	Not significant for OM grade	Yes—95% CI documented	Yes	Yes	Yes	1+
Xu et al. (2016)	No—PubMed, Cochrane, Elsevier, CNKI, VIP, CBM searched	Yes—Cochrane risk of bias tool	Yes	Significant benefit	Yes—95% CI documented	Yes	No	Yes	1−
Yang et al. (2019)	Yes	Yes—Cochrane risk of bias tool	Yes	Significant benefit	Yes—95% CI documented	Yes	No	Yes	1+
Münstedt et al. (2019)	Yes	Yes—Jada	N/A	Significant benefit	Descriptive	Yes	Yes	Yes	1−
Liu et al. (2019)	Yes	Yes—Cochrane risk of bias tool	No—different products used as control	Significant benefit	Yes—95% CI documented	Yes	No	Yes	1−
Oral glutamine									
Leung and Chan (2016)	Yes	Yes—Cochrane risk of bias tool	No—included 1 retrospective study	Significant benefit	Yes—95% CI documented	Yes	No	Yes	1+
Sayles et al. (2016)	No—only Medline searched	No—quality not assessed	N/A	Significant benefit	Descriptive	No—sample size small	Yes	Yes	1−

The overall methodological quality of three out of the four studies examining the use of BHM to mitigate RIOM was low. Only one study [12] had moderate methodological quality.

#### Honey

##### Primary outcome measure (oral mucositis measures)

Nine out of the twelve studies measured the incidence of severe OM. Seven of these studies [13–19] found statistically significantly fewer patients in the honey group had severe OM; the other two studies [20, 21] found no statistically significant difference between the groups. Four studies [16–18, 21] measured onset of OM. All found onset of OM was delayed in the honey group although only the first three calculated statistical significance. Two studies measured the mean OM grade; one study [13] found a statistically significant lower mean grade of OM during the second 3 weeks of RT; the other study [17] found a lower mean OM score but did not calculate the statistical significance. Two studies measured the difference in OM grade between the intervention and control groups. One study [22], a SR, reviewed 17 studies and found a lower OM grade in the honey group in 12 out of the 17 studies; the other study [19] found no statistically significant difference between the groups. One study [23] measured incidence of OM and found a statistically significant lower incidence of OM over the course of treatment. One study [19] measured the number of OM lesions and found statistically significantly fewer OM lesions in the honey group.

##### Secondary outcome measures

Six studies measured pain. Three of these studies [17, 19, 24] found statistically significantly lower pain scores in the honey group; one SR [22] reported that four out of the five studies it reviewed found lower pain scores in the honey groups; two studies [13, 20] found no statistically significant difference in pain scores between the groups.

Six studies measured weight loss. Four studies [13, 16, 17, 21] found statistically significant less weight loss in the honey groups; one SR [22] found less weight loss in the honey groups in the studies it reviewed, and one study [20] found no statistical significant difference between the groups.

Three studies [16, 21, 22] measured RT interruptions. All found fewer RT interruptions in the honey groups; the former two studies had statistically significant findings, and the latter did not calculate statistical significance.

Four studies measured QoL; three studies [17, 19, 22] found higher QoL scores in the honey group but only one of those [17] calculated the statistical significance. The fourth study [20] found no statistically significant difference between the groups.

Three studies recorded AEs: one study [20] found most of the participants who dropped out of the study reported nausea, a strong taste of honey or burning in the mouth. One study [13] reported AEs but it is more likely these were related to the OM itself rather than the product. One study [18] reported that there were no AEs related to honey.

##### Quality of studies

Only two out of the twelve studies [18, 21] investigating the use of honey to mitigate RIOM had moderate methodological quality. The other ten had low methodological quality.

#### Oral glutamine

##### Primary outcome measure (oral mucositis measures)

Seven out of the eight studies examining OG [25–31] measured incidence of severe OM. All but one study [25] found statistically significantly fewer patients in the OG group had severe OM. Five studies measured onset of OM. Three of these studies [26, 29, 30] found a statistically significant delay in onset of OM in the OG group; the other two studies [27, 32] found no statistically significant difference between the groups. Three studies [25, 27, 29] measured maximum OM scores with all finding statistically significantly lower maximum OM score in the OG groups. Three studies measured duration of OM; one study [26] found a statistically significant shorter duration of OM in the OG group; one study [27] found no statistically significant difference, and one SR [29] reviewed two studies, one of which found a statistically significant difference and the other did not. One study [32] measured incidence of OM (grades 1 to 4) and found no statistically significant difference between the groups. One study [27] measured mean OM and found a statistically significant lower mean OM score in the OG group during weeks 5 and 6 of RT. One study [32] measured functional OM and found no statistically significant difference between the groups.

##### Secondary outcome measures

Four studies measured pain; two of those studies [27, 29] found a statistically significant reduction in pain in the OG group; one study [30] found fewer participants in the OG group experienced pain although the statistical significance was not reported; one study [32] found no statistically significant difference between the groups.

Three studies measured the number of participants requiring analgesics. One of those studies [27] found no statistically significant difference; one SR [29] reviewed a study which found no difference; and one study [30] found fewer participants in the OG group required analgesics, although the statistical significance was not reported.

Three studies measured weight loss. One SR [29] reviewed two studies, one of which found statistically significantly less weight loss in the OG group and the other did not; one study [32] found no statistically significant difference between the groups; and one study [31] found statistically significantly less weight loss in the OG group.

Three studies [29–31] measured the number of participants requiring feeding tubes. All found fewer patients in the OG group required feeding tubes fitted, although only the former two reported that the findings were statistically significant.

Four studies recorded AEs. Three studies [27, 29, 32] reported no AEs related to OG. One study [30] reported more AEs in the control group, but it was likely these were related to OM rather than the product.

##### Quality of studies

Two studies [25, 28] had moderate methodological quality. The other six studies had low methodological quality.

##### Risk of bias across studies

The author considered that the risk of bias across the studies was high due to heterogeneity.

### Recommendations for clinical practice

The GRADE Evidence to Decision framework [8] was used to assess the evidence from this SR. A summary of the judgements and conclusions for interventions to mitigate RIOM in HNCPs are outlined in Tables 5 and 6.

**Table 5:** Evidence to Decision framework justifications

Criterion	Justification		
	BHM	Honey	OG
Is the problem a priority?	**Yes** RIOM can affect up to 100% of HNCPs patients having RT and so finding a suitable intervention is a priority.	**Yes** RIOM can affect up to 100% of HNCPs having RT, and so finding a suitable intervention is a priority.	**Yes** RIOM can affect up to 100% of HNCPs patients having RT, and so finding a suitable intervention is a priority.
How substantial are the desirable anticipated effects?	**Moderate** All four studies found BHM mitigated RIOM.	**Moderate** 11 out of 12 studies found honey mitigated RIOM.	**Moderate** 7 out of 8 studies found OG mitigated RIOM.
How substantial are the undesirable anticipated effects?	**Small** 1 study found poor tolerance to the product (burning mouth).	**Trivial** 1 study found poor tolerance to the product (burning mouth, nausea and disliking taste of Manuka honey).	**Trivial** 4 studies reported AE. None found any related to consumption of OG.
What is the overall certainty of the evidence of the effects?	**Low** 3 out of 4 studies were of low quality which affects certainty of evidence.	**Moderate** 2 out of 12 studies were of moderate methodological quality.	**Moderate** 2 out of the 8 studies were of moderate methodological quality.
Is there important uncertainty about or variability in how much people value the outcome?	**Possibly important uncertainty** Some patients may not value the intervention due to poor tolerance.	**Possibly important uncertainty** Some patients may not value the intervention due to poor tolerance to Manuka honey.	**Probably no important uncertainty or variability** No evidence of uncertainty to consuming product
Do the desirable effects outweigh the undesirable effects?	**Favours the comparison** Poor tolerance of product	**Favours the intervention** Poor tolerance to Manuka honey only	**Favours the intervention** No AE recorded
How large are the resource requirements?	**Moderate costs** Only interventions with low costs were examined in this SR and so resource requirements were low.	**Moderate costs** Only interventions with low costs were examined in this SR, and so resource requirements were low.	**Moderate costs** Only interventions with low costs were examined in this SR, and so resource requirements were low.
What is the certainty of the evidence of resource requirements?	**Very low** Resource requirements were not examined in any of the studies.	**Very low** Resource requirements were not examined in any of the studies.	**Very low** Resource requirements were not examined in any of the studies.
Are the net benefits worth the incremental cost?	**Probably favours the intervention** However, no cost-effect analysis was conducted.	**Probably favours the intervention** However, no cost-effect analysis was conducted.	**Probably favours the intervention** However, no cost-effect analysis was conducted.
What would be the impact on health equality?	**Probably no impact** It could be prescribed to NHS patients in the UK at no cost to them.	**Probably no impact** It is likely that patients would have to purchase this product which some patients may not be able to afford. So, alternative funding resources would need to be identified.	**Probably no impact** This product can be prescribed in UK to NHS patients at no cost to them.
Is the intervention acceptable to key stakeholders?	**No** Health care providers (HCPs) may be reluctant to prescribe due to AEs. All studies included patients having moderate-dose RT.	**Probably no** May only reduce complications of RIOM but not mitigate severity HCPs may not recommend to patients having higher doses of RT (3 out of 6 studies included patients having moderate-dose RT). HCP may not prescribe to diabetic patients.	**Probably yes** 2 studies included patients having IMRT; in one study, patients had shrinking field technique. However, one MA included patients having any RT technique and still found it efficacious.
Is the intervention feasible to implement?	**Yes** Implementation requires assessment of RIOM weekly from 1st week of RT to 1 month post RT Assessor training	**Yes** Implementation requires assessment of RIOM weekly from 1st week of RT to 1 month post RT Assessor training	**Yes** Implementation requires assessment of RIOM weekly from 1st week of RT to 1 month post RT Assessor training

**Table 6:** Evidence to Decision framework

Intervention	BHM	Honey	OG
Recommendation	Strong recommendation against the intervention	Conditional recommendation for the intervention	Strong recommendation for the intervention
Justification	Due to low-quality evidence of all studies reviewed and poor tolerance to product in one study	Eleven out of 12 studies found honey is effective at mitigating RIOM. Two of the studies had moderate methodological quality.	Six out of 8 studies found OG is effective at mitigating RIOM. Two of these studies had moderate methodological quality.
Sub-group considerations		Not recommended for diabetic patients Recommend with caution for patients on high-dose RT	
Implementation considerations		Training of staff required to assess OM	Training of staff required to assess OM
Monitoring and evaluation considerations		Weekly assessment of OM during RT and then monthly for 6 months	Weekly assessment of OM during RT and then monthly for 6 months
Research priorities		• Conduct large-sample, multi-centre studies • Ensure participant and staff blinding • Consensus on methodology including OM assessment tool, presentation of OM data, secondary outcome measures • Studies are conducted on patients having a high-dose of RT. • Data is analysed for each type of cancer treatment (RT alone, RT or CRT, or CRT only). • Optimum dose and frequencies of interventions are examined. • Identification of an effective placebo • Short- and long-term AEs are recorded and analysed. • Cost-effectiveness of the interventions is examined.	• Consensus on methodology including OM assessment tool, presentation of OM data, secondary outcome measures • Data is analysed for each type of cancer treatment (RT alone, RT or CRT, or CRT only). • Optimum dose and frequency of interventions are examined. • Short- and long-term AEs are recorded and analysed. • Cost-effectiveness of the interventions is examined.

Although the findings in the studies examining BHM were mainly positive, the author cannot recommend BHM to mitigate RIOM due to the overall low methodological quality and poor tolerance of the product.

Eleven out of the twelve studies examining honey found it to be efficacious either in reducing the incidence of severe OM or mean OM grade, or delaying onset of OM. Additionally, of those eleven studies, two were of moderate methodological quality. However, one of the studies with moderate methodological quality [21] found honey to be efficacious at delaying onset of OM (and reducing RT interruptions and weight loss) but not at reducing OM severity. Therefore, the author can only recommend honey to reduce complications of RIOM, but not to mitigate it. Three out of the six RCTs included patients having moderate doses of RT, and so the author cannot recommend this intervention for patients having higher doses of RT (at least 64 Gy). Additionally, there is a potential risk of honey consumption in diabetic patients. Finally, the author cannot recommend Manuka honey due to the poor tolerance.

Seven out of the eight studies examining the use of OG to mitigate RIOM had favourable findings. Two studies were of moderate methodological quality, and there were no adverse effects recorded. So, the author can recommend OG to mitigate RIOM.

## Discussion

This systematic review examined the efficacy of low-cost interventions to mitigate RIOM. The review identified interventions where there had been four or more studies examining it, conducted within the last 5 years. These interventions were BHM, honey and OG. The search identified twenty-four studies. The efficacies of the interventions were examined through the assessment of OM and secondary outcome measures. The review examined the interventions’ safety through the collection of data on adverse effects encountered. Following this, the evidence was applied to the GRADE EtD frameworks to inform clinical guidelines.

Recurrent themes that emerged included small sample sizes, most RCTs being single-centre studies, lack of blinding, heterogeneity, lack of data on AEs and lack of analysis of cost-effectiveness.

Most of the RCTs were small, single-centre studies, and even the two multi-centre studies had small sample sizes. Small samples are at risk of false-negative findings, and single-centre studies limit generalisability. Few of the studies examined in this review were blinded and those that were not risk bias. Also, in the blinded studies examining honey, the distinct taste and consistency of honey possibly increased the risk of performance bias. Identification of an effective placebo is necessary for well-conducted blinding and to reduce the risk of bias.

There was significant heterogeneity identified in the studies making it difficult to draw robust conclusions. Areas where heterogeneity was identified include OM assessment tools used, presentation of OM data, secondary outcome measures, doses and frequency of intake of the interventions, type of honey used, cancer treatments delivered (including patients receiving RT alone, CRT alone, or RT or CRT; type of RT machines; RT techniques—such as conventional or IMRT, and RT dose) and inclusion of certain cancer types. To reduce heterogeneity, the author recommends consensus of a methodology to be used in future studies.

Four OM assessment tools were identified. Although use of different OM assessment tools may introduce heterogeneity, one study [20] found good inter-reliability between RTOG, WHO and OMAS.

Overall, OM data was presented in twelve ways (for example data was presented as severity of OM, incidence of OM and onset of OM), and twenty-nine secondary outcomes were recorded, which introduced heterogeneity into the studies. The most common ways that OM data was presented was as incidence of severe OM and onset to OM; the most common secondary outcome measures used were pain, weight loss and RT interruptions. So, the author recommends that future studies present data in these ways and use the aforementioned secondary outcome measures.

The dose and frequency of consumption of the products varied which also introduced heterogeneity. The author recommends that studies examining the optimum dose be conducted. The type of honey used in the studies introduced further heterogeneity. One study [20] used Manuka honey, another [17] used thyme honey and the others used locally sourced, or pure, honey. Pooling data from studies using different types of honey may compromise the findings since some types of honey may be more effective at mitigating RIOM than others. One MA [21] found that the type of honey did not confound the findings; another MA [18] found local and pure natural honey efficacious and Manuka honey not efficacious at mitigating RIOM. An SR [22] reviewed thirteen studies which found conventional honey to be efficacious and four studies which found Manuka honey not to be efficacious.

Three BHM studies and three honey studies included participants having moderate doses of RT (between 50 and 64 Gy). It is likely that OM is less severe in patients having lower RT doses, and there is a possibility that including patients on lower doses makes the findings more favourable. Therefore, the findings can only be cautiously applied to patients having higher doses of RT.

There was additional heterogeneity due to inclusion of patients having different types of cancer treatment: either RT alone, CRT alone, or RT or CRT. Two studies [10, 13] found that BHM only mitigated RIOM in patients having RT alone, not in those having CRT; and two studies [18, 26] found the intervention efficacious for patients having either RT or CRT. Therefore, the author recommends future research examining the efficacy of the interventions for each cancer treatment option.

There was further heterogeneity in the types of radiotherapy delivered. Some studies included patients having treatment on cobalt machines, or conventional RT, where it is likely that RIOM is greater, due to larger margins required for the treatment field. Other studies included patients having intensity-modulated RT (IMRT) which treats smaller margins, and so RIOM is likely to be less severe. Two out of the six OG RCTs [25, 31] only included patients having IMRT. However, there were favourable results in the OG RCT [30] using conventional RT and in the OG MA [28], which had moderate methodological quality, which included patients having treatment using any type of RT technique. However, more research is needed to understand if RT techniques are confounding factors.

Although eight studies measured acute AEs [12, 13, 18, 20, 27, 29, 30, 32], none measured long-term AEs. One may assume that, due to the sugar content, prolonged consumption of honey can induce dental caries. However, a recent study [33] found that honey can prevent dental caries. The high sugar content makes honey unsuitable for long-term consumption by diabetic patients [34]. This contraindication was considered by four of the honey studies [13, 14, 16, 17] which excluded people with diabetes from participating, and another study [20] where participants were asked to monitor their blood sugar levels. However, excluding diabetic patients reduces generalisability of the findings. An RCT examining the use of parenteral alanyl-glutamine dipeptide, used as a supplement for autologous bone marrow transplant patients [35], found an increased mortality rate in the intervention group. However, a more recent SR and MA [36] reviewing glutamine supplementation for haematopoietic stem cell transplantation found no effect of either oral or IV glutamine on mortality rates. The author recommends future studies that examine the long-term AE of the interventions.

A significant limitation of the studies included in the SR was the quality of the methodologies. The methodologies of only five out of the twenty-four studies identified were of moderate quality. It is likely that the internal validity of low-quality studies may be compromised, and it is, therefore, difficult to draw robust conclusions. Therefore, it is recommended that future studies continue to improve the quality of the methodologies.

The focus of this review was to examine low-cost interventions to mitigate RIOM. Low-cost interventions were classed as those with few set-up costs. However, none of the studies examined the cost-effectiveness of the interventions, and so the author cannot make strong recommendations based on this. The author recommends future research in this area. The author acknowledges that there are financial barriers to producing high-quality research on low-cost interventions. Until more high-quality studies are available, the author recommends that clinicians consider the best available evidence-based interventions.

There were some limitations in the methodology of this SR. One limitation was that the search for studies was not comprehensive. The search only included studies in English language, those where the full text was available, and published studies. So, it is likely that selection and publication bias was present.

The author included interventions where there had been at least four studies conducted. Most studies examining RIOM have small sample sizes and are of low methodological quality and so risk false-negative findings. Including interventions with four or more studies reduces this risk, and so more robust conclusions could be drawn. However, including interventions where there had been fewer, good-quality studies may have been more appropriate. The author excluded studies conducted more than 5 years ago so that only the most up-to-date studies were included. However, selection bias could have been reduced by not limiting the search by year of publication.

Another limitation was that the SR only examined low-cost interventions. When making recommendations for clinical practice, the primary aim should be to find efficacious interventions over cost-saving ones. An alternative approach to research in this area could be to find cost-saving methods for already-established interventions for RIOM. For example, finding lower cost LLLT devices, finding ways to reduce training costs or having regional centres delivering LLLT (to reduce the number of devices needed and number of people trained to deliver the treatment).

A further limitation of this SR was that the research was conducted by one person, which may introduce bias. Finally, the SR only examined the efficacy of interventions to mitigate RIOM, and so conclusions cannot be applied to other causes of OM.

## Conclusion

The author cannot recommend the BHM to mitigate RIOM due to the low quality of the studies and poor tolerance to the product. The author cannot recommend honey to mitigate RIOM but can recommend it to reduce complications of RIOM (for example weight loss, pain, RT interruptions) for patients on moderate doses of RT but not for diabetic patients. The author can recommend OG to mitigate RIOM. There is a need for high-quality studies with a consensus of the methodology to reduce heterogeneity and examination of the cost-effectiveness of the interventions.
